# EpCAM+ Liver Cancer Stem‐Like Cells Exhibiting Autocrine Wnt Signaling Potentially Originate in Cirrhotic Patients

**DOI:** 10.1002/sctm.16-0248

**Published:** 2017-01-18

**Authors:** Ritu Khosla, Archana Rastogi, Gayatri Ramakrishna, Viniyendra Pamecha, Ashok Mukhopadhyay, Madavan Vasudevan, Shiv Kumar Sarin, Nirupma Trehanpati

**Affiliations:** ^1^ Department of Molecular and Cellular MedicineInstitute of Liver and Biliary SciencesNew DelhiIndia; ^2^ Department of PathologyInstitute of Liver and Biliary SciencesNew DelhiIndia; ^3^ Department of Liver Transplant and Hepato Pancreato Biliary SurgeryInstitute of Liver and Biliary SciencesNew DelhiIndia; ^4^ Department of HepatologyInstitute of Liver and Biliary SciencesNew DelhiIndia; ^5^ Stem Cell Biology LaboratoryNational Institute of ImmunologyNew DelhiIndia; ^6^ Bionivid Technology Pvt LtdBangaloreIndia

**Keywords:** Epithelial cell adhesion molecule, Cancer stem cells, Cirrhosis, Hepatocellular carcinoma, Wnt

## Abstract

Hepatocellular carcinoma (HCC) is believed to originate from cancer stem cells (CSCs). While epithelial cell adhesion molecule (EpCAM) is a marker of normal hepatic stem cells (HSCs), EpCAM+ cells from HCC behave like CSCs. Since HCC mostly develops on a cirrhotic background, we sought to determine whether CSC‐like EpCAM+ cells exist in patients with advanced cirrhosis. Both flow cytometry and immunohistochemistry showed that frequency of EpCAM+ cells in advanced cirrhosis was increased as compared to control. To determine whether increased EpCAM population in advanced cirrhosis harbors any CSC‐like cells, we compared molecular and functional features of EpCAM+ cells from advanced cirrhosis (Ep+CIR; *n* = 20) with EpCAM+ cells from both HCC (Ep+HCC; *n* = 20) and noncancerous/noncirrhotic (control) (Ep+NSC; *n* = 7) liver tissues. Ep+CIRs displayed similarity with Ep+HCC cells including upregulated expression of stemness and Notch pathway genes, enhanced self‐renewal in serial spheroid assay and generation of subcutaneous tumors in nonobese diabetic/severe combined immunodeficiency mice. Moreover, transcriptome and miRNome of Ep+CIRs appeared closer to that of Ep+HCC cells than Ep+NSCs. Interestingly, more than 50% micro RNAs (miRNAs) and transcripts specifically expressed in Ep+HCCs were also expressed in Ep+CIRs. However, none of Ep+NSC specific miRNAs and only 7% Ep+NSC specific transcripts were expressed in Ep+CIRs. Further, according to gene expression and in vitro Wnt inhibition analysis, autocrine Wnt signaling appeared to be a distinct feature of Ep+CIR and Ep+HCC cells, which was absent from Ep+NSCs. EpCAM+ cells in advanced cirrhosis possibly include a population of CSC‐like cells which can be explored for early diagnosis of HCC development. Stem Cells Translational Medicine *2017;6:807–818*

**Significance Statement**

Hepatocellular carcinoma (HCC) frequently occurs in the background of cirrhosis and is believed to originate from cancer stem cells (CSCs). In this study, CSC like EpCAM+ cells were observed in patients with advanced cirrhosis. These cells had the potential to form tumors because of their limitless self‐renewal through autocrine activation of Wnt signaling. Targeting these cells at initial stages of cirrhosis can help to prevent progression towards HCC, which is often detected at a stage that is not amenable to present treatment modalities.

## Introduction

Hepatocellular carcinoma (HCC) is the fifth most common cancer and second leading cause of cancer related deaths globally 1. Despite therapeutic advancements in the treatment of HCC survival rates are poor due to presentation of the disease at advanced stage 2. Recent studies indicate that HCC arises from liver cancer stem cells (CSCs) leading to tumor heterogeneity 3. The origin of HCC in over 80% cases is in the milieu of chronic liver injury, mainly cirrhosis 4. In cirrhosis, hepatocytes lose their replicative potential, resulting in the activation of normal hepatic stem cells (HSCs) 5. Active division in presence of increased reactive oxygen species and inflammation, makes HSCs amenable to incorporate mutations and convert into CSCs, which eventually can lead to the development of HCC 6. Thus, identification of CSCs can be of help in early diagnosis of HCC development in advanced cirrhotic patients.

Intriguingly, surface markers like epithelial cell adhesion molecule (EpCAM), CD133, CD90, CD13, and OV6 are used to identify HSCs as well as CSCs, making it difficult to differentiate between them 7. Of these markers, EpCAM (MUC1) is an adhesion molecule commonly used to identify normal HSCs. It is present in developing as well as adult livers and is absent from the mature hepatocytes 8, 9. However, in HCC, EpCAM expression correlates with poor patient survival 10. Further, EpCAM+ cells isolated from alphafetoprotein positive (AFP+) HCC tissues have been demonstrated to behave like CSCs 11, 12. Accordingly, we chose EpCAM as a marker for identification of normal stem cells (NSCs) from control and CSCs from AFP+ HCC liver tissues.

While existence of CSCs has been well demonstrated in HCC, their characterization in cirrhotic patients remains elusive, with only few reports addressing this concept 13, 14. We observed an increase in the number of EpCAM+ cells during advanced cirrhosis, warranting further studies to delineate if they have any similarity with CSCs. Since normal HSCs and CSCs express common surface markers, only certain functional characteristics can help in differentiating between them. For instance, Notch and Wnt signaling pathways are involved in the self‐renewal and differentiation of normal HSCs, but, their constitutive activation has been implicated in CSCs 15, 16, 17. In the present study, we therefore compared molecular and functional features of EpCAM+ cells from advanced cirrhosis, HCC and paired adjacent noncancerous/noncirrhotic (control) liver tissues along with EpCAM‐cells from the same HCC tissues (Ep‐HCC). Functional comparison was performed by checking the in vitro spheroid formation in nonadherent culture conditions and in vivo tumor formation in nonobese diabetic/severe combined immunodeficiency (NOD/SCID) mice. Next generation sequencing (NGS) was used to compare micro RNA (miRNA) as well as mRNA profiling in all the cell groups. NGS revealed that Wnt signaling was regulated by genes and miRNAs commonly expressed by EpCAM+ cells of both advanced cirrhosis and HCC. Owing to its well‐known role in CSCs, we validated the key genes of the Wnt pathway in a larger cohort. Further, the effect of Wnt inhibitor was evaluated to determine whether any cell group is exhibiting autocrine Wnt signaling.

## Patients and Methods

### Patient Samples

Liver tissues were obtained from patients undergoing Living Donor Liver Transplant (LDLT) for advanced cirrhosis (*n* = 20), and LDLT/resection for HCC (*n* = 20), at the Institute of Liver and Biliary Sciences (New Delhi, India) during 2012–2014 years. Histologically normal appearing liver tissues (noncancerous/noncirrhotic region adjacent to tumor portion) were also taken from patients having HCC on a noncirrhotic background and served as controls (*n* = 7). Patients between 18–80 years of age were enrolled in the study. Only HCC tissues that were AFP+ were included. Patients with alcohol, nonalcoholic steatohepatitis, hepatitis B virus (HBV) and hepatitis C virus (HCV) related HCC or advanced cirrhosis were included, while those having acute liver failure, acute‐on‐chronic liver failure or any viral coinfections including Human Immunodeficiency Virus (HIV)‐HBV, HIV‐HCV and HBV‐HCV were excluded from the study. This study was approved by the Institutional Ethics Committee and informed consent was obtained from each patient or their close relatives for inclusion in the study. About 35–40 gm of liver tissues from the patients were processed and subjected to different analyses as detailed in Supporting Information Figure 1.

### Immunohistochemistry

Histopathological analyses were performed on 5 µm thick formalin fixed paraffin embedded liver tissue sections using standard pathology laboratory protocols. In brief, sections were deparaffinized with xylene and rehydrated through graded alcohol (100, 90, 80, and 70%). Sections were then stained with Hematoxylin and eosin (H&E). Reticulin staining was performed to distinguish HCC from cirrhosis as per the standard protocol 18.

For immunohistochemistry (IHC), after rehydration, sections were treated with 3% H_2_O_2_ followed by antigen retrieval with sodium citrate buffer (pH = 6.0) and blocking with 20% goat serum. Primary anti‐human EpCAM, Hepatocyte Specific Antigen (HSA) or Glypican‐3 antibodies were used at a dilution of 1:250, 1:500, and 1:500, respectively, for staining (Santa Cruz Biotechnologies, CA, USA, https://www.scbt.com). CD133 and CD90 antibodies were procured from Abnova, Taiwan, http://www.abnova.com and used at a dilution of 1:500 and 1:200, respectively. Detection was performed using SuperSensitive Polymer‐HRP IHC Detection System/DAB large volume kit (BioGenex, CA, USA, https://store.biogenex.com), in accordance with the manufacturer's instructions. The sections were counterstained with hematoxylin to demonstrate the nuclei and were observed under the microscope. Semiquantification of EpCAM expression was defined by Quick (Q) scoring system. Q scores were calculated by multiplying the percentage of positive cells with staining intensity (0 = no, 1 = weak, 2 = moderate, and 3 = strong staining) to yield a score ranging from 0 to 300.

### Enrichment of EpCAM+ Cells Using Magnet Based Cell Separation

The cell isolation procedure from liver tissues was performed by magnetic based assay with some modifications 19. In brief, tissue explants were washed with phosphate buffer saline (PBS) and chopped into small pieces. After digestion with 0.05% type IV collagenase (Worthington Biochemical Corporation, NJ, USA, http://www.worthington-biochem.com) for 30 minutes at 37°C, tissues were again minced and the cell suspension was passed through a 70‐µm cell strainer (BD Biosciences, NJ, USA, https://www.bdbiosciences.com) to remove tissue debris. Red blood cells (RBCs) were lysed by 1x RBC lysis buffer (Stem Cell Technologies, Vancouver, Canada, https://www.stemcell.com) and the cells were washed again with PBS. An aliquot of the single cell suspension was used to determine the percentage of EpCAM+ cells by flow cytometry. Viable and nonviable cells were counted by trypan blue staining and then used to isolate EpCAM+ cells by magnet assisted cell sorting. In brief, depending on the cell number, they were labeled with appropriate amount of EpCAM+ selection cocktail using EpCAM+ Cell Isolation Kit (Stem Cell Technologies, Vancouver, Canada), according to manufacturer's instructions. Purity of EpCAM+ and EpCAM‐sorted cells were evaluated by flow cytometry.

### Flow Cytometric Analysis

Cells were stained with anti‐human EpCAM PE (eBiosciences, CA, USA, http://www.ebioscience.com/) antibody for 20 minutes at RT. After washing with PBS, cells were acquired on BD FACS Calibur (BD Biosciences, San Jones, CA) and data was analyzed using Flow Jo software version 7.5.

### Quantitative Reverse‐Transcription Polymerase Chain Reaction

Total RNA was extracted from the sorted cells' pellet using MIRVana kit (Ambion, TX, USA, https://www.thermofisher.com/in/en/home/brands/invitrogen/ambion.html). The concentration of RNA was measured using Nanodrop ND‐1000 spectrophotometer (ThermoFisher Scientific, DE, USA, http://www.thermofisher.com/in/en/home.html) and quality was checked by bioanalyzer (Agilent Technologies, CA, USA, www.agilent.com). The RNA samples (∼50 ng) were then amplified using a random hexamer primer to form complimentary DNA.

Quantitative reverse‐transcription polymerase chain (qRT‐PCR) reaction was performed with a SYBR Green PCR Kit (Applied Biosystems, DE, USA, https://www.thermofisher.com/in/en/home/brands/applied-biosystems.html) using an ABI PRISM 7700 Sequence Detector and ViiA 7 software (Applied Biosystems, DE, USA). The primers of selected genes were designed using Primer 3 software **(**Supporting Information Table 1**)**. The gene expression level was normalized against 18S RNA (control gene). Subsequently, the relative gene expression values were determined using log of 2^−ΔΔCT^.

### Spheroid Assay

Sorted cells obtained after magnetic based cell separation were suspended in 0.9% methyl cellulose in knockout Dulbecco's modified Eagle medium/nutrient mixture F‐12 (DMEM/F12) media, containing 10% knockout serum replacement, 1% Penicillin‐Streptomycin‐Amphotericin B, 20 ng/ml human recombinant Epidermal Growth Factor and 10 ng/ml human recombinant Fibroblast Growth Factor (Life Technologies, CA, USA, https://www.thermofisher.com/in/en/home/brands/invitrogen.html) and seeded in a six‐well ultralow attachment plate (Corning Inc., Corning, NY, USA, www.corning.com) at a density of about 5,000 cells per well. Plates were incubated at 37°C and 5% CO_2_, for 12–14 days, during which fresh media was added every third day. When most of the spheroids attained at least 50 µm diameter, they were washed with PBS, trypsinized and repassaged under same culture conditions. Spheroids were maintained for a total of six passages. After each passage the size and number of spheroids was recorded.

### Animal Studies

Twenty‐five thousand or 50,000 freshly isolated EpCAM+ cells were resuspended in 200 µl of a 1:1 mixture of DMEM/F12:Matrigel (BD Biosciences, NJ, USA) and subcutaneously injected into 6 to 8 weeks old male nonobese diabetic/severe combined immunodeficiency (NOD/SCID) mice (The Jackson Laboratories, CA, USA, https://www.jax.org/). For each cell type (Ep+NSC, Ep+CIR, Ep+HCC, and Ep‐HCC), three mice were injected with three different patient samples. Formation of tumor was observed till 8–10 weeks after injection of cells. Tumor volume was calculated by using the formula ½[(largest diameter)^2^ × smallest diameter]. The experimental protocol was approved by Institutional Animal Ethics Committee (IAEC 298/12), National Institute of Immunology, India. All animals were treated with humane care. They were kept in individual ventilated cages and fed with autoclaved acidified water and irradiated food ad libitum.

### Next Generation Sequencing for Small RNA and mRNA

Detailed protocol is given in Patients and Methods section of Supporting Information annexure.

### Wnt Inhibitor Assay

EpCAM+ cells were plated at a density of 10,000 cells per well in ultra‐low attachment plates with IWP12 (Inhibitor of Wnt production compound 12) (Sigma‐Aldrich, Tokyo, Japan, www.sigmaaldrich.com/) at a concentration of 5 µM per well. Spheroid formation was compared in terms of both size and number with corresponding cells plated without the inhibitor.

### Statistical Analysis

Data were analyzed using the statistical software Prism (version 5; GraphPad Software) and are reported as mean ± standard error mean (SEM). For analysis of the clinical data, Kruskal‐Wallis Test followed by probability adjustment by Mann‐Whitney test was performed. Here *p* ≤.017 was considered significant. For other experiments the unpaired Student's *t* test was performed to compare two mean values and *p* values <.05 were considered statistically significant.

## Results

### Clinicopathological Features of Patients

The demographic profile of all the subjects enrolled in this study is shown in Table 1. Out of total patients with advanced cirrhosis and/or HCC, 20% were affected due to excessive alcohol intake and another 20% with HBV infection. Only one patient had HCV related HCC. Remaining 57% patients had unknown etiology. Male to female ratio in patients with advanced cirrhosis or HCC was same. There was no significant difference in the AFP levels of cirrhotic and HCC patients due to high standard deviation. Out of 20 cirrhotic patients, only one patient (5%) had AFP above the threshold value of 10 ng/ml. In case of HCC as well, only 20% had AFP values above the threshold. AST and ALT levels in both the groups were also similar. The serum bilirubin and International normalized ratio (INR) levels were higher in cirrhotic patients as compared to HCC patients and so were the MELD scores.

**Table 1:** Demographic profile of the study group patients

Parameters	Advanced cirrhosis (*n* = 20) (A)	HCC (*n* = 20) (B)	*p* value
Age (years)	47.17 ± 10.94	57.2 ± 11.75	.129
Sex (M:F)	04:01	04:01	—
Creatinine (mg/dl)	0.78 ± 0.29	0.78 ± 0.19	1
INR	1.64 ± 0.33	1.22 ± 0.25	.004
Bilirubin (mg/dl)	3.64 ± 3.03	1.72 ± 2.07	.012
AST (IU/L)	51.92 ± 23.26	79 ± 45.3	.87
ALT (IU/L)	29.5 ± 15.7	63.4 ± 35.7	.77
AFP (ng/ml) (>10 ng/ml)	4.2 ± 1.51 (5)	136.3 ± 273.8 4 (20)	.16
MELD Score	19.6 ± 7	8.9 ± 2.9	<.000
Etiology			
HBV	4 (20)	7 (35)	—
ALD	6 (30)	2 (10)	—
HCV	0 (0)	1 (5)	—
Cryptogenic	10 (50)	10 (50)	—

All values are expressed as mean ± SD or number (%); *p* < .017 = significant.

Abbreviations: —: no data; AFP: alfafetoprotein; ALD: alcoholic liver disease; ALT: alanine aminotransferase; AST: aspartate transaminase; HBV: Hepatitis B virus; HCV: Hepatitis C virus; INR: International Normalized Ratio; M:F, male:female; MELD: model for end stage liver disease.

### Histopathological Characterization of the Tissue Specimen

Histologically normal, cirrhotic and HCC liver tissues used in the present study were initially validated by a pathologist. HCC occurred in the background of cirrhosis or in the absence of cirrhosis as depicted in the gross pictures (Supporting Information Fig. 2A). Histologically normal liver tissues obtained from noncancerous/noncirrhotic region adjacent to tumor were classified on the basis of no significant appearance of fibrosis and single chord arrangement of benign hepatocytes in HE sections (Supporting Information Fig. 2B). Cirrhotic tissues were composed of regenerative parenchyma (Supporting Information Fig. 2C). HE staining of HCC specimen showed the characteristic trabecular and pseudoglandular architecture (Supporting Information Fig. 2D). Malignant hepatocytes were polygonal cells displaying varying degree of nuclear enlargement and atypia. Reticulin staining is a silver impregnation technique used to delineate reticulin fibers and is therefore useful in distinguishing HCC (which is reticulin deficient due to thick chord arrangement of cells) from cirrhosis. Reticulin staining revealed a reticulin poor HCC in contrast to preserved reticulin staining of cirrhotic liver tissue specimen (Supporting Information Fig. 2E). IHC for HCC specific surface markers HSA and Glypican‐3 further highlighted the tumor (Supporting Information Fig. 2F). Since, EpCAM+ cells from AFP+ HCC tissues have been shown to behave like CSCs 11, for subsequent experiments we used HCC tissues which showed AFP positivity in IHC (Supporting Information Fig. 2G).

### Increased Expression of EpCAM in Cirrhotic Liver Tissues

**Figure 1:**
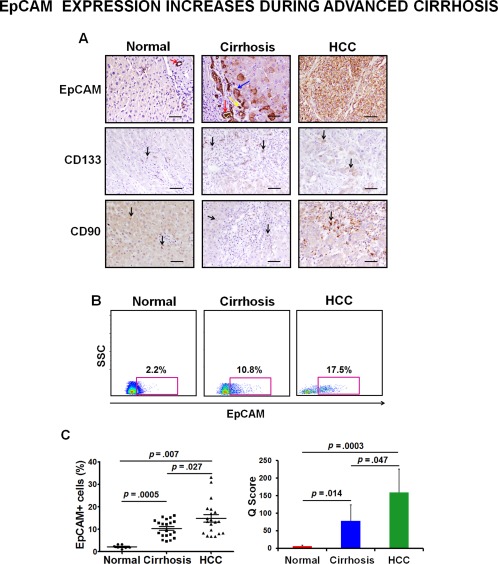
Advanced cirrhosis show increased expression of EpCAM+ cells**. (A)**: Representative photomicrographs for immunohistochemical (IHC) analysis of EpCAM, CD133, and CD90 (brown) on paraffin‐embedded sections of histologically normal (*n* = 7), advanced cirrhotic (*n* = 20), and HCC (*n* = 20) liver tissues. Slides were counterstained with hematoxylin (x200; Scale bars = 100 µm). **(B)**: Representative dot plots for flowcytometric analysis of total liver cells from same patient groups with antihuman phycoerithrin‐EpCAM antibody. **(C)**: Scatter plot is representing the percentage of EpCAM+ cells in each patient as determined by flow cytometry (left panel). Quantitation of IHC data is represented as a bar graph (right panel). *p* < .05 = significant. Abbreviations: EpCAM, epithelial cell adhesion molecule; HCC, hepatocellular carcinoma.

We compared the expression of some of the most commonly used surface markers for CSC identification namely EpCAM, CD133, and CD90 in the respective tissue sections by IHC (Fig. 1A). A very weak expression of CD133 was observed in the control and cirrhotic liver sections confined to the hepatic progenitor cells. Within the HCC sections as well, weak to moderate cytoplasmic staining of CD133 was observed. In accordance with the published reports 20, CD90 expression was mainly detected in inflammatory and endothelial cells. There was not much difference in the level of CD90 expression in control and cirrhotic liver tissue. The HCC section showing maximum expression of CD90 is represented in the figure, although most of the other sections showed weak or no expression. The expression of EpCAM was detected mainly in bile ductules of control liver tissues and was completely absent from mature hepatocytes. IHC in cirrhosis revealed that EpCAM expression was increased particularly near the periportal areas in the bile ductules, oval cells, and intermediate hepatocytes. HCC tissues showed strong membranous staining of EpCAM. Flow cytometric analysis also showed an increase in number of EpCAM+ cells in advanced cirrhosis as compared to histologically normal tissue specimens (Fig. 1B). However, quantitation of both flow cytometric and IHC data demonstrated that the frequency of EpCAM+ cells was highest in HCC tissues (Fig. 1C). These results clearly indicate an increase in the EpCAM expression during progression toward HCC. However, to establish the presence of CSCs in the increased EpCAM population of cirrhotic liver tissues, their molecular and functional characterization was performed as described in the following sections.

### EpCAM+ Cells from Cirrhotic Livers Display Enhanced Self‐Renewal

**Figure 2:**
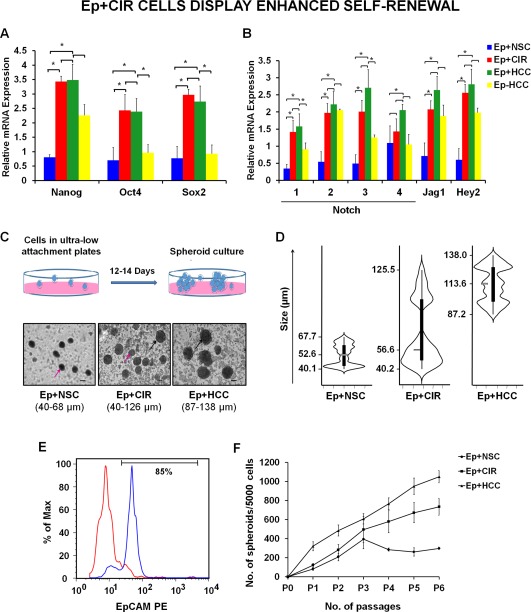
EpCAM+ cells from advanced cirrhosis display enhanced self‐renewal. **(A, B)**: Expression of stemness and Notch signaling genes in sorted Ep+NSC (*n* = 5), Ep+CIR (*n* = 20), Ep+HCC (*n* = 20), and Ep‐HCC (*n* = 20) cells determined by quantitative reverse transcription‐polymerase chain reaction. Results are expressed as mean of log 2^‐ΔΔCt^ ± standard error. (*, *p* < .05). **(C)**: EpCAM+ cells from normal, advanced cirrhosis and HCC formed spheroids under nonadherent culture conditions (*n* = 5 in each group) (Images from first passage, x200). **(D)**: Spheroids from Ep+CIR were heterogeneous in size as represented by two lobes in the Violin plot. Vertical axis is showing the range and mean size of the spheroids (*n* = 3 in each group). **(E)**: Flow cytometric analysis of single cells from the spheroids showed enrichment of EpCAM+ cells in them. **(F)**: After every 14–16 days, spheroids were trypsinized to form single cells and 5,000 cells per well were re‐plated with fresh medium till six passages. Number of spheroids in each sample was counted after every passage and plotted in a graph (*n* = 3 in each group) (values are represented as mean ± SEM). Abbreviations: Ep+CIR, EpCAM+ cells from advanced cirrhosis; Ep+HCC, EpCAM+ cells from HCC, Ep+NSC, EpCAM+ cells from noncancerous/noncirrhotic (control); HCC, hepatocellular carcinoma; NSCs, normal stem cells; Oct4, octamer‐binding transcription factor 4; Sox2, sex determining region Y‐box 2.

Deregulation of various signaling pathways has been implicated in maintaining self‐renewal of CSCs 21. For instance, Notch signaling promotes proliferation of CSCs. Further, *Nanog, Oct4, and Sox2* are key regulators of stemness involved in carcinogenesis 22, 23, 24. Interestingly, these stemness genes are direct targets of EpCAM 25. We therefore wanted to compare the level of expression of these genes in EpCAM+ cells isolated from control (Ep+NSC), advanced cirrhosis (Ep+CIR) and AFP+ HCC tissues (Ep+HCC). The purity of the sorted EpCAM+ cells was between 85 and 90% (Supporting Information Fig. 3). Expressions of *Nanog, Oct4, and Sox2* and Notch signaling genes (*Notch 1, 2, 3, Jag1, and Hey2*) were significantly higher (*p* < .05) in Ep+CIRs and Ep+HCCs in comparison to Ep+NSC **(**Fig. 2A, 2B, Supporting Information Table 2). However, there was no significant difference in the expression of these genes between Ep+CIRs and Ep+HCCs. Further, *Notch 4* showed no significant difference in the expression between all the three study groups. To monitor the validity of sorted Ep+HCCs, the expression of all the above genes was also analyzed in Ep‐HCC cells. All the genes showed decreased expression in Ep‐HCCs as compared to Ep+HCC cells.

Self‐renewal of CSCs can be established in vitro by serial spheroid assay 26. We checked the spheroid forming ability of EpCAM+ cells by growing them in nonadherent serum free culture. A striking difference was observed in the size of the spheroids formed by Ep+NSC, Ep+CIR, and Ep+HCC cells **(**Fig. 2C**)**. Violin plot clearly demonstrated that Ep+CIR spheroids were varied in size (40–126 µm). While most of them were smaller in size like Ep+NSC (40–68 µm), a small proportion were bigger like Ep+HCC (87–138 µm) spheroids **(**Fig. 2D**)**. This observation affirms the existence of a heterogeneous population of EpCAM+ cells within advanced cirrhotic liver tissues. Flow cytometric analysis revealed the enrichment of EpCAM+ cells within the spheroids, thereby demonstrating their clonal expansion **(**Fig. 2E**)**.

The spheroids were cultured for six passages. Number of spheroids formed by EpCAM+ cells isolated from both HCC and advanced cirrhosis increased exponentially at every passage. On the contrary, number of spheroids from Ep+NSCs increased till third passage following which it attained a stationary phase (Fig. 2F). Taken together, these results suggest that EpCAM+ cells from advanced cirrhosis have limitless self‐renewal capacity unlike Ep+NSCs.

### Ep+CIR Cells Possess Tumor Forming Ability

**Figure 3:**
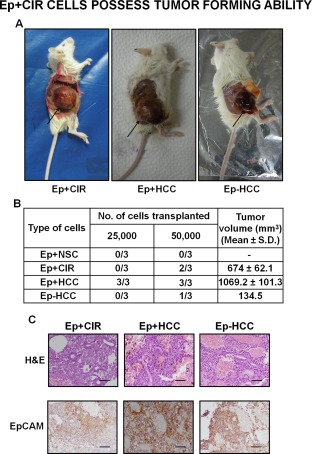
EpCAM+ cells from advanced cirrhosis possess tumor forming ability. **(A)**: Representative images of tumors derived from nonobese diabetic/severe combined immunodeficiency mice induced by Ep+CIR, Ep+HCC, and Ep‐HCC, after 8 weeks of subcutaneous injection (50,000 cells per mice) (*n* = 3 in each group). **(B)**: Table showing volume (mean ± SD) and incidence of tumor formation with different cell types (*n* = 3 in each group). **(C)**: Histopathological characterization by HE staining (upper panel) and immunohistochemical analysis of EpCAM expression in tumors derived by each cell type (lower panel) (×200; Scale bars = 100µm). (Lines highlighting width of thick trabeculae; Rays indicating pseudoglandular structure; Thick arrows pointing toward intense membranous staining of EpCAM in malignant hepatocytes). (*n* = 3 in each group). Abbreviations: EpCAM, epithelial cell adhesion molecule; Ep+CIR, EpCAM+ cells from advanced cirrhosis; Ep+HCC, EpCAM+ cells from HCC, Ep+NSC, EpCAM+ cells from noncancerous/noncirrhotic (control); H&E, Hematoxylin and eosin; HCC, hepatocellular carcinoma; NSCs, normal stem cells.

The gold standard for testing CSCs is their ability to generate tumors upon injection into mice 27, 28. We subcutaneously injected 25,000 and 50,000 Ep+NSC, Ep+CIR, Ep+HCC, and also Ep‐HCC cells in flanks of NOD/SCID mice. Ep+HCC cells formed tumors in all the three mice upon transplantation of 25,000 cells/mice (Fig. 3A). Ep+CIR cells also generated subcutaneous tumors albeit when higher cell number was used, while no tumor formation was observed with 25,000 cells **(**Fig. 3A, 3B**)**. Ep+NSCs did not generate any tumor even with 50,000 cells/mice whereas EpCAM‐ HCC cells formed a very small tumor in one out of three mice injected with 50,000 cells. Tumor nodules formed by each cell type were confirmed by histopathological examination. Representative images shown in Figure 3C reveal characteristic histomorphological features of HCC with presence of thick trabeculae surrounded by sinusoidal cell along with focal pseudoglandular arrangement of malignant hepatocytes. These cells display enlarged nucleus with high nucleocytoplasmic ratio, conspicuous nucleoli and moderate to abundant eosinophilic cytoplasm. All tumor sections showed strong membranous immunostaining for EpCAM.

The low frequency of tumor formation with cirrhotic EpCAM+ cells cannot be fully explained by number of injected cells alone. It may be affected by experimental artefacts like viability and purity of the injected cells. Further, the percentage of CSCs within the total EpCAM+ cell population from cirrhotic liver is expected to be very low and might vary from patient to patient. Nevertheless, it is reasonable to conclude from this result that EpCAM+ cells from advanced cirrhosis include a subset of CSCs.

### Ep+CIR Cells Carry Ep+HCC Cell Like Signatures in Advanced Cirrhotic Livers

In order to decipher the similarity between Ep+HCC and Ep+CIR at the molecular level, NGS was performed in Ep+HCC, Ep+CIR, Ep+NSC, and Ep‐HCC cell populations. Deep sequencing of transcripts and small RNAs resulted in an average of 15 million reads per sample for small RNome and 72 million reads per sample for transcriptome (Supporting Information Fig. 4A, 4B). Unique Tag distribution of small RNAs in Ep+NSCs was significantly higher as compared to the other three groups of cells (Supporting Information Fig. 4C). A total of 24,196 transcripts and 389 miRNAs were detected to be expressed collectively in the four samples (as per the criteria mentioned in the methods).

#### Ep+HCC Cell Specific miRNA Signatures Seen in Ep+CIR Cells

**Figure 4:**
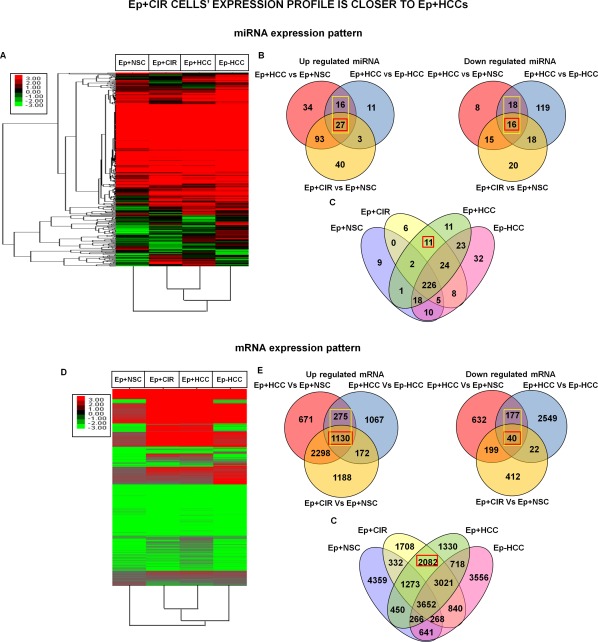
miRNome and transcriptome analysis shows close association between Ep+CIR and Ep+HCC cells. **(A)**: Unsupervised hierarchical cluster analysis of differentially expressed miRNAs illustrating close association of Ep+CIR with Ep+HCC cells. **(B)**: Venn diagram showing that significant number of miRNAs differentially expressed in Ep+HCC versus Ep+NSC and Ep‐HCC (Yellow box) are similarly differentially expressed in Ep+CIR versus Ep+NSC (Red Box). **(C)**: Ep+CIRs express a significant number of Ep+HCC specific miRNAs (11/22). **(D)**: Unsupervised hierarchical cluster analysis of differentially expressed mRNAs in different group of cells. **(E)**: Venn diagram showing that significant number of mRNAs differentially expressed in Ep+HCC (yellow box) are similarly differentially expressed in Ep+CIR versus Ep+NSC (Red Box). **(F)**: Ep+CIR express a significant number of Ep+HCC specific transcripts (2082/3412). Red, black, and green cells in the heat maps depict high, no and low expression levels respectively, as indicated by the scale bar. Abbreviations: EpCAM, epithelial cell adhesion molecule; Ep+CIR, EpCAM+ cells from advanced cirrhosis; Ep+HCC, EpCAM+ cells from HCC, Ep+NSC, EpCAM+ cells from noncancerous/noncirrhotic (control); HCC, hepatocellular carcinoma; NSCs, normal stem cells.

Unsupervised hierarchical clustering of the differentially expressed miRNAs clearly showed that Ep+CIRs and EP+HCC cells clustered together while Ep‐HCC and Ep+NSC miRNAs did not largely overlap with Ep+CIR (Fig. 4A).

In order to obtain CSC related miRNAs, Ep+HCC miRNome was compared with Ep+NSC and Ep‐HCC (< or >2‐fold and *p* < .05). Further, to determine CSC related miRNAs in advanced cirrhosis, Ep+CIR cells were compared with Ep+NSCs and together the number of differentially expressed miRNAs in the three comparisons was plotted in one Venn diagram. Twenty seven out of 43 upregulated (63%) and 16 out of 34 downregulated (47%) miRNAs in Ep+HCC were also similarly differentially expressed in Ep+CIR (Fig. 4B). Interestingly, 11 out of 22 Ep+HCC specific miRNAs (141, 18a, 511, 561, 548o, 200a, 200b, 224, 31, 7‐1, and 210) were also expressed in Ep+CIR cells but not in Ep+NSCs and Ep‐HCCs (Fig. 4C; Supporting Information Table 3). However, none of the Ep+NSC specific miRNAs were expressed in Ep+CIR.

#### Ep+HCC Specific Gene Signature in Advanced Cirrhosis

In parallel with the deep sequencing based miRNA studies, we also investigated the global changes in gene expression patterns associated with Ep+NSC, Ep+HCC, Ep+CIR, and Ep‐HCC cell populations. Unsupervised hierarchical clustering of the differentially expressed transcripts reiterated the observation that Ep+CIR profile was closer to that of Ep+HCC as compared to Ep+NSC and Ep‐HCC cells, indicating the presence of dominant molecular signature of CSCs during advanced cirrhosis (Fig. 4D).

Venn diagram depicting the comparison of Ep+HCC with Ep+NSC and Ep‐HCC, and Ep+CIR with Ep+NSC showed that 1,130 upregulated and 40 down regulated genes in Ep+HCCs were also similarly differentially expressed in Ep+CIRs (Fig. 4E). A significant number of transcripts (2082) were specifically expressed only in Ep+CIR and Ep+HCC (Fig. 4F; Supporting Information Table 4), which together with 11 Ep+HCC and Ep+CIR specific miRNAs, could be a representative signature of Ep+HCCs in the cirrhotic liver. On the contrary, very few transcripts (332) were expressed exclusively in both Ep+NSC and Ep+CIR cells.

Taken together, the results clearly indicate that Ep+CIRs are similar to Ep+HCCs and are distinct from Ep+NSC cells at the molecular level.

#### Pathways Regulated by miRNAs/Transcripts Commonly Expressed by Ep+CIR and Ep+HCC Cells

**Figure 5:**
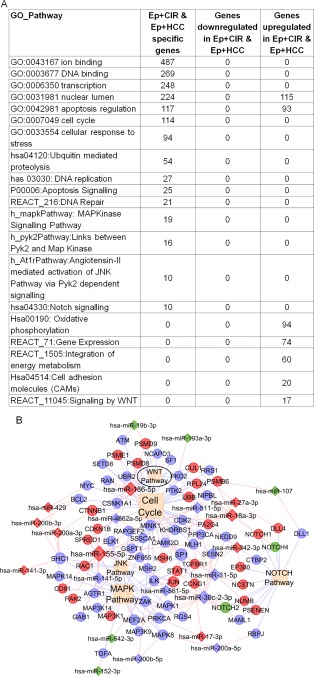
Wnt signaling is regulated by miRNAs and transcripts commonly expressed by EpCAM+ cells of cirrhosis and HCC. **(A)**: Top twenty pathways regulated by miRNAs and transcripts commonly expressed by EpCAM+ cells of cirrhosis and HCC. **(B)**: Network analysis of relevant pathways regulated by miRNAs and transcripts commonly expressed in Ep+CIR and Ep+HCC cells. Triangles represent miRNAs, circles represent genes and diamond represents pathways. Red color indicates upregulation, green color indicates downregulation and blue indicates Ep+CIR and Ep+HCC specific expression Wnt signaling (encircled in black) is an important pathway implicated in CSCs. Abbreviations: EpCAM, epithelial cell adhesion molecule; Ep+CIR, EpCAM+ cells from advanced cirrhosis; Ep+HCC, EpCAM+ cells from HCC, Ep+NSC, EpCAM+ cells from noncancerous/noncirrhotic (control); HCC, hepatocellular carcinoma; NSCs, normal stem cells.

miRNAs and genes that were (a) specifically expressed only in Ep+HCC and Ep+CIR, (b) commonly upregulated in Ep+HCC and Ep+CIR, and (c) commonly downregulated in Ep+HCC and Ep+CIR, were subjected to significant gene ontology and pathway analysis to identify important biological events regulated by them (Fig. 5A). Wnt, Notch, mitogen‐activated protein kinase, and c‐Jun N‐terminal kinase (JNK) were some of the important pathways regulated by commonly expressed miRNAs and transcripts of Ep+CIR and Ep+HCCs (Fig. 5B). Thorough literature survey also identified same molecular pathways involved in regulation of CSCs.

#### EpCAM+ Cells of Advanced Cirrhosis Patients Exhibit Autocrine Wnt Signaling

Among the commonly expressed pathways, Wnt signaling was of particular interest due to its role in stem cell self‐renewal and cancer development. Further, Yamashita et al. recently reported that Wnt/β‐catenin signaling augments self‐renewal and inhibits the differentiation of liver CSCs by the expression of the stem cell marker EpCAM 11. Therefore, we chose to validate the expression of Wnt pathway genes in EpCAM+ cells from control, advanced cirrhotic and HCC tissues.

**Figure 6:**
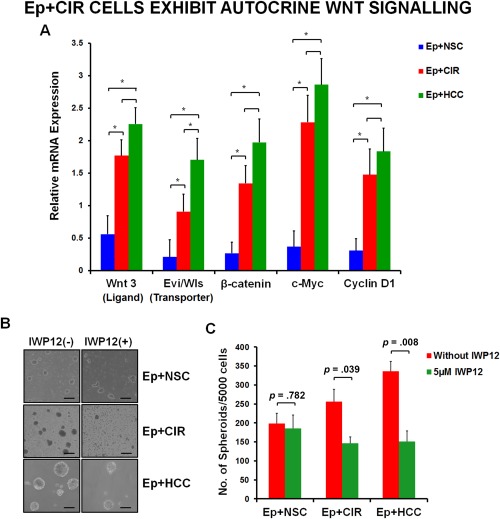
EpCAM+ cells from advanced cirrhosis and HCC exhibit autocrine Wnt signaling**. (A)**: Real time analysis showed >2‐fold upregulation of Wnt pathway genes including e*vi/wls* (Wnt transporter) in both Ep+CIR (*n* = 20) and Ep+HCC (*n* = 20) as compared to Ep+NSCs (*n* = 5). Results are expressed as mean of log 2^−ΔΔCt^ ± standard error. (*, *p* < .05). **(B, C)**: In the presence of IWP12 (an inhibitor of Wnt acyl‐transferase), spheroids formed by EpCAM+ cells of both advanced cirrhosis and HCC were reduced in size as well as number. There was no significant effect of the inhibitor on spheroid formation of Ep+NSCs (x200; Scale bars = 100µm) (*n* = 3 in each group). (*p* < .05 = significant). Abbreviations: EpCAM, epithelial cell adhesion molecule; Ep+CIR, EpCAM+ cells from advanced cirrhosis; Ep+HCC, EpCAM+ cells from HCC, Ep+NSC, EpCAM+ cells from noncancerous/noncirrhotic (control); HCC, hepatocellular carcinoma; NSCs, normal stem cells.

Signaling molecules of Wnt pathway were >2‐fold upregulated in Ep+CIR and Ep+HCC compared to Ep+NSC cells **(**Fig. 6A**)**, particularly *Wnt3*, which is a ligand in canonical Wnt signaling. It is reported that the secretion of Wnt proteins require Evi/Wls (Evenness interrupted/Wntless), a multipass transmembrane protein. It acts as a cargo receptor for Wnt proteins, shuttling them from the Golgi to the plasma membrane where they act in an autocrine or paracrine manner to activate Wnt signaling pathways 29. Therefore, we also checked the expression of *Evi/Wls* in the three study groups. Although highest in HCC, the expression of *Evi/Wls* was significantly increased in EpCAM+ cells from advanced cirrhotic patients than controls (Fig. 6A), indicating the existence of autocrine Wnt signaling in them. In addition, a significant increase in expression of *β‐catenin* and two of its well‐known targets *c‐Myc* and *Cyclin D1*, was also noted in both Ep+CIR and Ep+HCC cells.

Chen et al. in 2009 reported that IWP compounds act on porcupine, a member of the membrane‐bound O‐acyltransferase (MBOAT) family and inhibit palmitoylation of Wnt proteins, which is essential for their secretion and signaling ability 30. Therefore, we cultured the EpCAM+ cells from advanced cirrhosis and HCC patients in ultra‐low attachment plates with and without the Wnt inhibitor, IWP12. The number and size of spheroids formed in the presence of IWP12 was significantly reduced with Ep+CIR and Ep+HCC cells when compared to corresponding EpCAM+ cells grown without the inhibitor **(Fig.** 6 **B,** 6 **C**). The number of spheroids formed was reduced by 44% and 54%, respectively. The responsiveness to Wnt inhibition confirmed that both Ep+CIR and Ep+HCCs exhibit autocrine Wnt signaling unlike the Ep+NSCs. Cumulatively, the observation that Ep+CIR cells showed increased expression of *Evi/Wls* and *Wnt3* and were even responsive to Wnt inhibitor, IWP12, re‐emphasized their molecular similarity to Ep+HCCs.

## Discussion

HCC frequently occurs in the background of cirrhosis and is believed to originate from CSCs 31. Cirrhosis is marked by regenerative nodules resulting from localized proliferation of hepatocytes in response to liver injury. These nodules can eventually progress to become dysplastic nodules or HCC 32. In chronic liver injury, ductular reactions become prominent due to suppressed hepatocyte proliferation, leading to the activation of stem cell response 33, 34. EpCAM+ cells have been reported as immediate progeny of the HSCs, formed due to ductular reactions 35. In accordance with these reports, we too observed increased expression of EpCAM+ cells near the ductular regions of advanced cirrhosis liver sections. An increase in the EpCAM expression together with increased rate of cellular proliferation could possibly dictate the fate toward transformation into CSCs. Existence of CSCs during cirrhosis was earlier addressed by Sun et al. However, they used the complete cirrhotic tissue to show its tumorigenicity and did not characterize it at cellular level 13. More recently in a mouse model, a collagenase resistant cell population was identified within cirrhotic livers which could generate tumors in mice with chronic damage and compensatory proliferation in their liver. These cells were shown to overexpress EpCAM and were called as HCC progenitor cells 14. The present study, to our knowledge, is the first to report the existence of EpCAM+ cancer stem‐like cells in advanced cirrhotic patients. All the patients included as advanced cirrhosis in this study had undergone liver transplantation.

Using molecular and functional parameters we determined CSC like traits in Ep+CIRs. Notch is a well‐studied pathway implicated in the regulation of both normal HSCs and CSCs. Notch signaling was activated in HSCs during biliary regeneration, but was restricted during hepatocellular regeneration 36. On the contrary, a previous study indicated increased Notch activity in CSCs as compared to HSCs in breast cancer 37. Cirrhotic patients recruited in this study had primary hepatocellular injury rather than primary cholangiocyte injury (Primary Biliary Cirrhosis and Primary Sclerosing Cholangitis patients were not enrolled in the study). Yet, we observed that *Jag1, Notch 1*, *Notch 2*, *Notch 3,* and *Hey2* were significantly upregulated in both Ep+CIR and Ep+HCC cells as compared to Ep+NSCs, indicating the appearance of CSC like phenotype during advanced cirrhosis.

It is well documented that self‐renewal capacity of a cell can be quantified by spheroid formation in vitro, in particular by analyzing the number and size of cells within the spheroid and the ongoing generation (self‐renewal) of spheroids through repeated passages. 38. Ep+CIR cells formed few large sized spheroids compared to the majority of smaller ones, providing direct evidence for the presence of a heterogeneous population of EpCAM+ cells within the cirrhotic livers. Increased spheroid formation over repeated passages reflected the limitless proliferation capabilities of Ep+CIR cells. Further, unlike Ep+NSCs, Ep+CIR cells were capable of generating tumors when injected subcutaneously in NOD/SCID mice. Ep+CIR cells thus exhibited enhanced self‐renewal with uncontrolled proliferation and tumor formation, all of which are hallmark features of a CSC 39.

In order to determine the similarity at the molecular level between Ep+CIR and Ep+HCC cells, NGS was performed. Sequencing data deciphered that JNK, MAPK and Wnt signaling were some of the important pathways regulated by miRNA and genes commonly shared by Ep+CIR and Ep+HCC cells. JNKs were earlier considered to have pro‐apoptotic functions in response to stress, inflammatory or oncogenic signals 40. However, emerging evidence now suggests that the JNKs, especially JNK1, play an important role in the malignant transformation of cells and in tumorigenesis 41. Studies have also shown the association of higher JNK1 activation with a poorer prognosis as well as overexpression of EpCAM, CD24, CD133, KRT19, and AFP in HCC 42. Indeed, we too observed an over‐expression of JNK1 in Ep+CIR and Ep+HCC cells as compared to Ep+NSC (data not shown).

Interestingly, 11 out of 22 Ep+HCC specific miRNAs were also expressed in Ep+CIRs. Among these, miR‐511 is negatively associated with overall survival of HCC patients 43. miR‐210 promotes migration and invasion of HCC cells 44. miR‐141 has been reported to confer resistance to 5‐flurouracil in HCC cells 45. Importantly, miR‐18a and 224 have been proposed as serum biomarkers for diagnosis of HCC 46, 47. Thus, most of the 11 miRNAs are associated with imparting tumorigenic properties in HCC cells and their increased expression in Ep+CIRs reflects their transformed status. However, role of these miRNAs in CSCs still needs to be elucidated. Additionally, the functions of miR‐548o, 561, and miR‐7‐1 have not yet been well explored in context of both HCC and CSCs.

Wnt signaling is an important proliferation pathway, well documented in the pathogenesis of many cancers. Importantly, Yamashita et al. showed that Wnt/β‐catenin enriches the EpCAM+ CSCs by enhancing their self‐renewal and inhibiting their differentiation 48. We therefore validated the expression of Wnt pathway genes by qRT‐PCR and found them to be upregulated in both Ep+CIR and Ep+HCC cells. Colon CSCs have been shown to demonstrate autocrine Wnt secretion via Evi/Wls, a transporter of Wnt 49. Present data showed significantly increased expression of *evi* and *wnt3* in Ep+CIR and Ep+HCC cells. Subsequently, in the presence of Wnt inhibitor (IWP 12), the spheroid forming ability of both group of cells reduced significantly. To the best of our knowledge, these results report for the first time, the existence of autocrine Wnt signaling in EpCAM+ CSCs from HCC. In addition, presence of autocrine Wnt signaling in Ep+CIR strengthen our notion of existence of CSC like cells during advanced cirrhosis.

## Conclusion

In conclusion, we report the existence of a small population of EpCAM+ CSC like cells in advanced cirrhosis, which have the potential to develop into HCC because of their limitless self‐renewal through autocrine activation of Wnt signaling. Targeting these cells at initial stages of advanced cirrhosis can help to prevent progression toward HCC, which is often detected at a stage that is not amenable to present treatment modalities. However, to target these cells at cirrhotic stage, we need to identify an efficient biomarker which is preferentially expressed in CSCs and not NSCs. Further investigations of CSCs specific miRNAS in patient serum samples may highlight their use as biomarkers for early diagnosis of HCC at cirrhotic stage.

## Authors Contributions

R.K.: collection of patient samples, laboratory experiments, data acquisition, analysis and interpretation, statistical analysis, manuscript writing; A.R.: interpretation of immunohistochemistry data; V.P.: provision of transplant/resected liver tissue specimens and intellectual inputs; A.M.: provision of animal house facility, supervision of animal experiments and revision of manuscript; M.V.: acquisition and analysis of NGS data; G.R.: critical intellectual inputs and manuscript editing; S.K.S.: critical review of the concept, experimental designs and revision of the article; N.T.: conceptual design of study and critical revision of the article for important intellectual content.

## Disclosure of Potential Conflicts of Interest

The authors indicate no potential conflicts of interest.

## Supporting information

Supporting Information Figures.

Supporting Information Tables.

Supporting Information Table 4.

Supporting Information.

## References

[1] Chua HH , Tsuei DJ , Lee PH et al. RBMY, a novel inhibitor of glycogen synthase kinase 3beta, increases tumor stemness and predicts poor prognosis of hepatocellular carcinoma. Hepatology 2015;62:1480–1496. 26185016 10.1002/hep.27996

[2] Cance WG , Stewart AK , Menck HR. The National Cancer Data Base Report on treatment patterns for hepatocellular carcinomas: Improved survival of surgically resected patients, 1985‐1996. Cancer 2000;88:912–920. 10679662 10.1002/(sici)1097-0142(20000215)88:4<912::aid-cncr23>3.0.co;2-t

[3] Sell S. Cellular origin of hepatocellular carcinomas. Semin Cell Dev Biol 2002;13:419–424. 12468242 10.1016/s1084952102001295

[4] El‐Serag HB. Hepatocellular carcinoma. N Engl J Med 2011;365:1118–1127. 21992124 10.1056/NEJMra1001683

[5] Roskams T. Liver stem cells and their implication in hepatocellular and cholangiocarcinoma. Oncogene 2006;25:3818–3822. 16799623 10.1038/sj.onc.1209558

[6] Alison MR , Islam S , Lim S. Stem cells in liver regeneration, fibrosis and cancer: The good, the bad and the ugly. J Pathol 2009;217:282–298. 18991329 10.1002/path.2453

[7] Liu LL , Fu D , Ma Y et al. The power and the promise of liver cancer stem cell markers. Stem Cells Dev 2011;20:2023–2030. 21651381 10.1089/scd.2011.0012

[8] Turner R , Lozoya O , Wang Y et al. Human hepatic stem cell and maturational liver lineage biology. Hepatology 2011;53:1035–1045. 21374667 10.1002/hep.24157 PMC3066046

[9] Schmelzer E , Zhang L , Bruce A et al. Human hepatic stem cells from fetal and postnatal donors. J Exp Med 2007;204:1973–1987. 17664288 10.1084/jem.20061603 PMC2118675

[10] Ji J , Yamashita T , Budhu A et al. Identification of microRNA‐181 by genome‐wide screening as a critical player in EpCAM‐positive hepatic cancer stem cells. Hepatology 2009;50:472–480. 19585654 10.1002/hep.22989 PMC2721019

[11] Yamashita T , Ji J , Budhu A et al. EpCAM‐positive hepatocellular carcinoma cells are tumor‐initiating cells with stem/progenitor cell features. Gastroenterology 2009;136:1012–1024. 19150350 10.1053/j.gastro.2008.12.004 PMC2828822

[12] Terris B , Cavard C , Perret C. EpCAM, a new marker for cancer stem cells in hepatocellular carcinoma. J Hepatol 2010;52:280–281. 20006402 10.1016/j.jhep.2009.10.026

[13] Sun YL , Yin SY , Xie HY et al. Stem‐like cells in hepatitis B virus‐associated cirrhotic livers and adjacent tissue to hepatocellular carcinomas possess the capacity of tumorigenicity. J Gastroenterol Hepatol 2008;23:1280–1286. 18466286 10.1111/j.1440-1746.2008.05342.x

[14] He G , Dhar D , Nakagawa H et al. Identification of liver cancer progenitors whose malignant progression depends on autocrine IL‐6 signaling. Cell 2013;155:384–396. 24120137 10.1016/j.cell.2013.09.031 PMC4015514

[15] Dontu G , Jackson KW , McNicholas E et al. Role of Notch signaling in cell‐fate determination of human mammary stem/progenitor cells. Breast Cancer Res 2004;6:R605–615. 15535842 10.1186/bcr920 PMC1064073

[16] Wang Z , Li Y , Banerjee S et al. Emerging role of Notch in stem cells and cancer. Cancer Lett 2009;279:8–12. 19022563 10.1016/j.canlet.2008.09.030 PMC2699045

[17] Pannuti A , Foreman K , Rizzo P et al. Targeting Notch to target cancer stem cells. Clin Cancer Res 2010;16:3141–3152. 20530696 10.1158/1078-0432.CCR-09-2823 PMC3008160

[18] Yao S , Zhang J , Chen H et al. Diagnostic value of immunohistochemical staining of GP73, GPC3, DCP, CD34, CD31, and reticulin staining in hepatocellular carcinoma. J Histochem Cytochem 2013;61:639–648. 23686365 10.1369/0022155413492771 PMC3753886

[19] Ho DW , Yang ZF , Yi K et al. Gene expression profiling of liver cancer stem cells by RNA‐sequencing. PLoS One 2012;7:e37159. 22606345 10.1371/journal.pone.0037159 PMC3351419

[20] Yamashita T , Honda M , Nakamoto Y et al. Discrete nature of EpCAM+ and CD90+ cancer stem cells in human hepatocellular carcinoma. Hepatology 2013;57:1484–1497. 23174907 10.1002/hep.26168 PMC7180389

[21] Takebe N , Miele L , Harris PJ et al. Targeting Notch, Hedgehog, and Wnt pathways in cancer stem cells: Clinical update. Nat Rev Clin Oncol 2015;12:445–464. 25850553 10.1038/nrclinonc.2015.61 PMC4520755

[22] Takahashi K , Yamanaka S. Induction of pluripotent stem cells from mouse embryonic and adult fibroblast cultures by defined factors. Cell 2006;126:663–676. 16904174 10.1016/j.cell.2006.07.024

[23] Wen J , Park JY , Park KH et al. Oct4 and Nanog expression is associated with early stages of pancreatic carcinogenesis. Pancreas 2010;39:622–626. 20173672 10.1097/MPA.0b013e3181c75f5e

[24] Muller M , Hermann PC , Liebau S et al. The role of pluripotency factors to drive stemness in gastrointestinal cancer. Stem Cell Res 2016;16:349–357. 26896855 10.1016/j.scr.2016.02.005

[25] Lu TY , Lu RM , Liao MY et al. Epithelial cell adhesion molecule regulation is associated with the maintenance of the undifferentiated phenotype of human embryonic stem cells. J Biol Chem 2010;285:8719–8732. 20064925 10.1074/jbc.M109.077081 PMC2838295

[26] Pastrana E , Silva‐Vargas V , Doetsch F. Eyes wide open: A critical review of sphere‐formation as an assay for stem cells. Cell Stem Cell 2011;8:486–498. 21549325 10.1016/j.stem.2011.04.007 PMC3633588

[27] Gilbert CA , Ross AH. Cancer stem cells: Cell culture, markers, and targets for new therapies. J Cell Biochem 2009;108:1031–1038. 19760641 10.1002/jcb.22350 PMC2909872

[28] Hindriksen S , Bijlsma MF. Cancer stem cells, EMT, and developmental pathway activation in pancreatic tumors. Cancers 2012;4:989–1035. 24213498 10.3390/cancers4040989 PMC3712732

[29] Bartscherer K , Pelte N , Ingelfinger D et al. Secretion of Wnt ligands requires Evi, a conserved transmembrane protein. Cell 2006;125:523–533. 16678096 10.1016/j.cell.2006.04.009

[30] Chen B , Dodge ME , Tang W et al. Small molecule‐mediated disruption of Wnt‐dependent signaling in tissue regeneration and cancer. Nat Chem Biol 2009;5:100–107. 19125156 10.1038/nchembio.137 PMC2628455

[31] Yamashita T , Wang XW. Cancer stem cells in the development of liver cancer. J Clin Invest 2013;123:1911–1918. 23635789 10.1172/JCI66024 PMC3635728

[32] Coleman WB. Mechanisms of human hepatocarcinogenesis. Curr Mol Med 2003;3:573–588. 14527088 10.2174/1566524033479546

[33] Falkowski O , An HJ , Ianus IA et al. Regeneration of hepatocyte ‘buds’ in cirrhosis from intrabiliary stem cells. J Hepatol 2003;39:357–364. 12927921 10.1016/s0168-8278(03)00309-x

[34] Clouston AD , Powell EE , Walsh MJ et al. Fibrosis correlates with a ductular reaction in hepatitis C: Roles of impaired replication, progenitor cells and steatosis. Hepatology 2005;41:809–818. 15793848 10.1002/hep.20650

[35] Yoon SM , Gerasimidou D , Kuwahara R et al. Epithelial cell adhesion molecule (EpCAM) marks hepatocytes newly derived from stem/progenitor cells in humans. Hepatology 2011;53:964–973. 21319194 10.1002/hep.24122

[36] Boulter L , Govaere O , Bird TG et al. Macrophage‐derived Wnt opposes Notch signaling to specify hepatic progenitor cell fate in chronic liver disease. Nat Med 2012;18:572–579. 22388089 10.1038/nm.2667 PMC3364717

[37] Harrison H , Farnie G , Howell SJ et al. Regulation of breast cancer stem cell activity by signaling through the Notch4 receptor. Cancer Res 2010;70:709–718. 20068161 10.1158/0008-5472.CAN-09-1681 PMC3442245

[38] Dontu G , Abdallah WM , Foley JM et al. In vitro propagation and transcriptional profiling of human mammary stem/progenitor cells. Genes Dev 2003;17:1253–1270. 12756227 10.1101/gad.1061803 PMC196056

[39] Al‐Hajj M , Clarke MF. Self‐renewal and solid tumor stem cells. Oncogene 2004;23:7274–7282. 15378087 10.1038/sj.onc.1207947

[40] Liu J , Lin A. Role of JNK activation in apoptosis: A double‐edged sword. Cell Res 2005;15:36–42. 15686625 10.1038/sj.cr.7290262

[41] Durbin AD , Somers GR , Forrester M et al. JNK1 determines the oncogenic or tumor‐suppressive activity of the integrin‐linked kinase in human rhabdomyosarcoma. J Clin Invest 2009;119:1558–1570. 19478459 10.1172/JCI37958 PMC2689127

[42] Chang Q , Chen J , Beezhold KJ et al. JNK1 activation predicts the prognostic outcome of the human hepatocellular carcinoma. Mol Cancer 2009;8:64. 19686584 10.1186/1476-4598-8-64 PMC2732591

[43] Zhang J , Chong CC , Chen GG et al. A seven‐microRNA expression signature predicts survival in hepatocellular carcinoma. PLoS One 2015;10:e0128628. 26046780 10.1371/journal.pone.0128628 PMC4457814

[44] Ying Q , Liang L , Guo W et al. Hypoxia‐inducible microRNA‐210 augments the metastatic potential of tumor cells by targeting vacuole membrane protein 1 in hepatocellular carcinoma. Hepatology 2011;54:2064–2075. 22144109 10.1002/hep.24614

[45] Shi L , Wu L , Chen Z et al. MiR‐141 activates Nrf2‐dependent antioxidant pathway via down‐regulating the expression of Keap1 conferring the resistance of hepatocellular carcinoma cells to 5‐fluorouracil. Cell Physiol Biochem 2015;35:2333–2348. 25896253 10.1159/000374036

[46] Li L , Guo Z , Wang J et al. Serum miR‐18a: A potential marker for hepatitis B virus‐related hepatocellular carcinoma screening. Dig Dis Sci 2012;57:2910–2916. 22865399 10.1007/s10620-012-2317-y

[47] Okajima W , Komatsu S , Ichikawa D et al. Circulating microRNA profiles in plasma: Identification of miR‐224 as a novel diagnostic biomarker in hepatocellular carcinoma independent of hepatic function. Oncotarget 2016;7:53820–53836. 10.18632/oncotarget.10781 PMC5288224 27462777

[48] Yamashita T , Budhu A , Forgues M et al. Activation of hepatic stem cell marker EpCAM by Wnt‐beta‐catenin signaling in hepatocellular carcinoma. Cancer Res. 2007;67:10831–10839. 18006828 10.1158/0008-5472.CAN-07-0908

[49] Voloshanenko O , Erdmann G , Dubash TD et al. Wnt secretion is required to maintain high levels of Wnt activity in colon cancer cells. Nature Commun 2013;4:2610. 24162018 10.1038/ncomms3610 PMC3826636

