# Supplementary material for: EpCAM+ Liver Cancer Stem‐Like Cells Exhibiting Autocrine Wnt Signaling Potentially Originate in Cirrhotic Patients
Source: Stem Cells Transl Med. 2017 Jan 18;6(3):807–18. doi: 10.1002/sctm.16-0248 (PMC5442787; doi:10.1002/sctm.16-0248)
Supplement: Supplementary file 1 — Supporting Information Figures. [file SCT3-6-0807-s001.doc]

**SUPPORTING INFORMATION FIGURE LEGENDS**

**
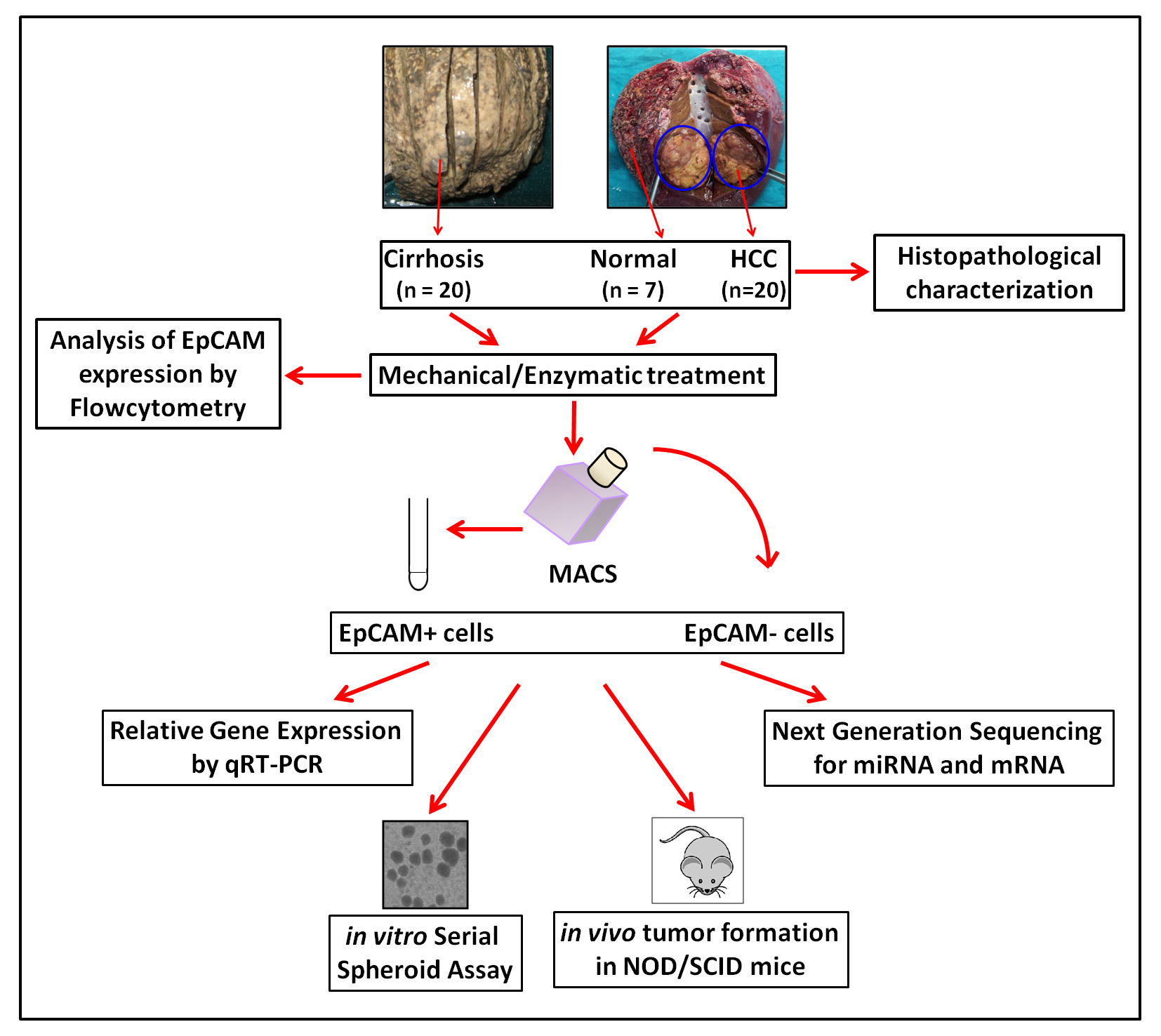
**

**Supporting Information Fig. 1. Schematic of the study design**

**
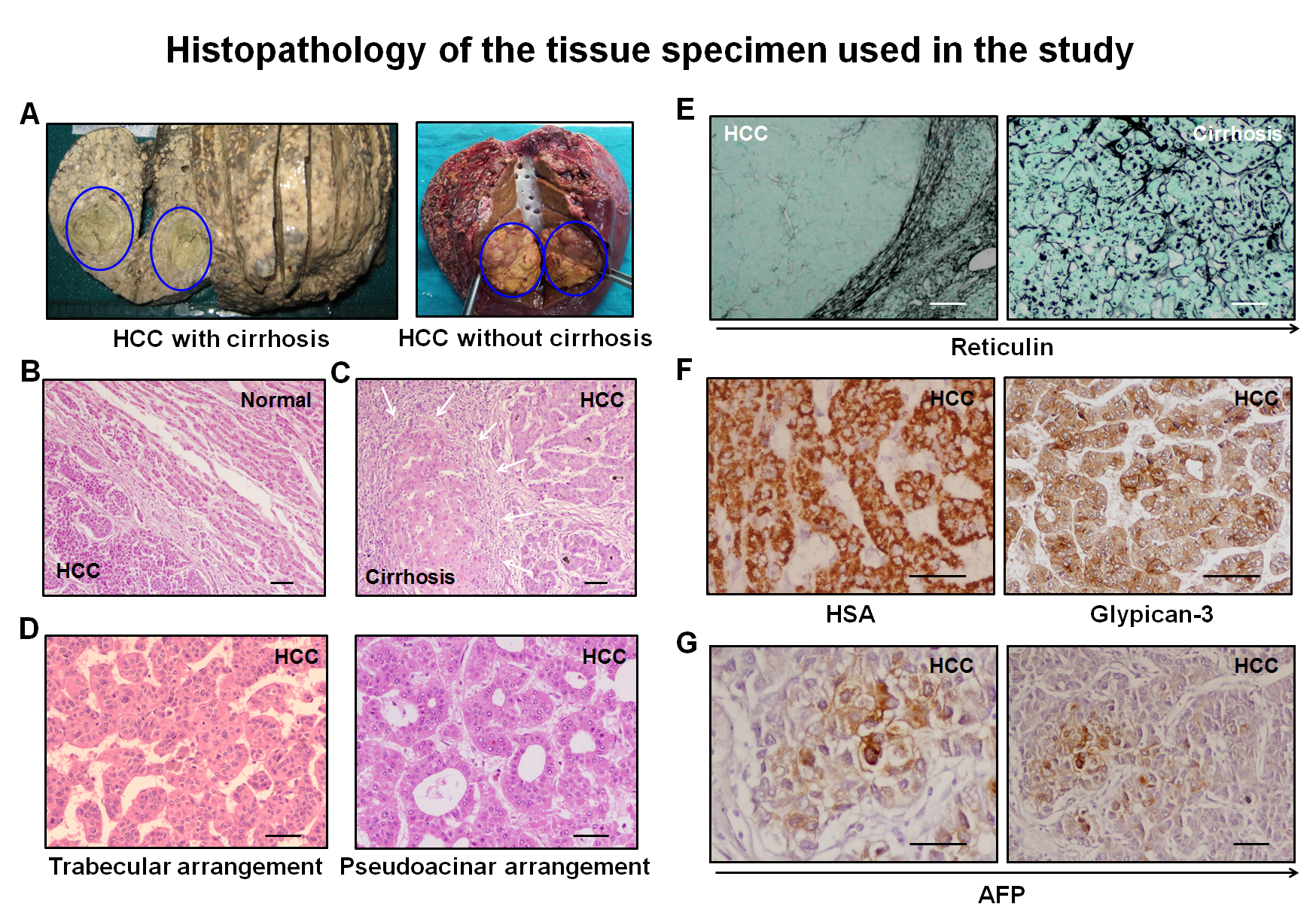
**

**Supporting Information Fig. 2. Histopathological features of the tissue specimen.** (A) Gross liver specimen showing HCC on a cirrhotic and a non-cirrhotic background. (B) HE showing no apparent fibrosis in non-cirrhotic/non-cancerous region adjacent to the tumor. (100x) (C) Cirrhotic tissue composed of regenerative parenchyma and surrounded by fibrous septa. (100x) (D) HE of the tumor tissue highlighting trabecular and pseudoacinar arrangement of tumor cells. (200x) (E) Reticulin staining differentiating HCC (Reticulin poor) from cirrhosis (Reticulin preserved). (200x) (F) Confirmation of HCC by immunohistochemical HSA and Glypican-3 staining. Slides were counterstained with hematoxylin (400x) (G) Immunohistochemical staining of HCC sections with AFP shown at 400x (left) and 200x (right). Slides were counterstained with hematoxylin to reveal the nuclei. (Scale bars = 100µm)


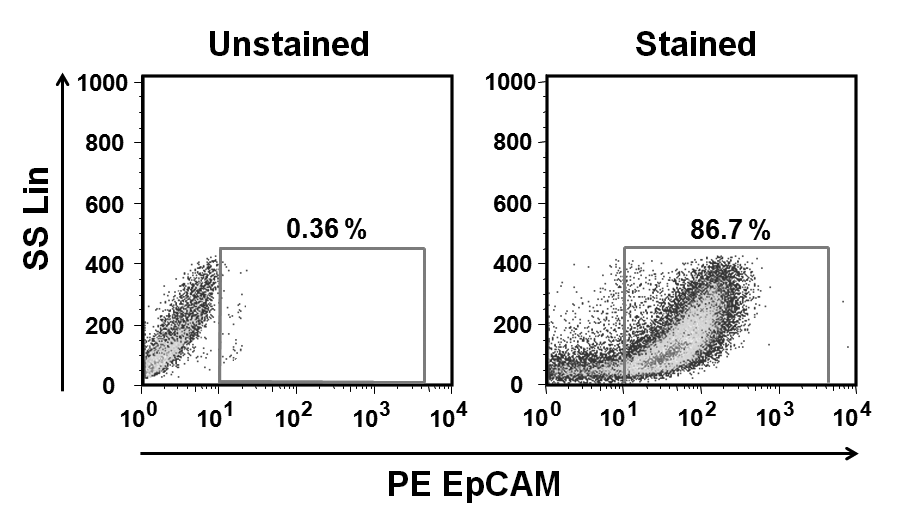


**Supporting Information Fig. 3. Purity of EpCAM+ cell population.** Representative flowcytometric dot plot of EpCAM+ cells isolated by magnetic separation from total liver cells showing the purity of the sorted fraction. Cells were stained with antihuman-EpCAM PE antibody and analysed by BD-FACS Calibur using FlowJo Software version 7.5.

**
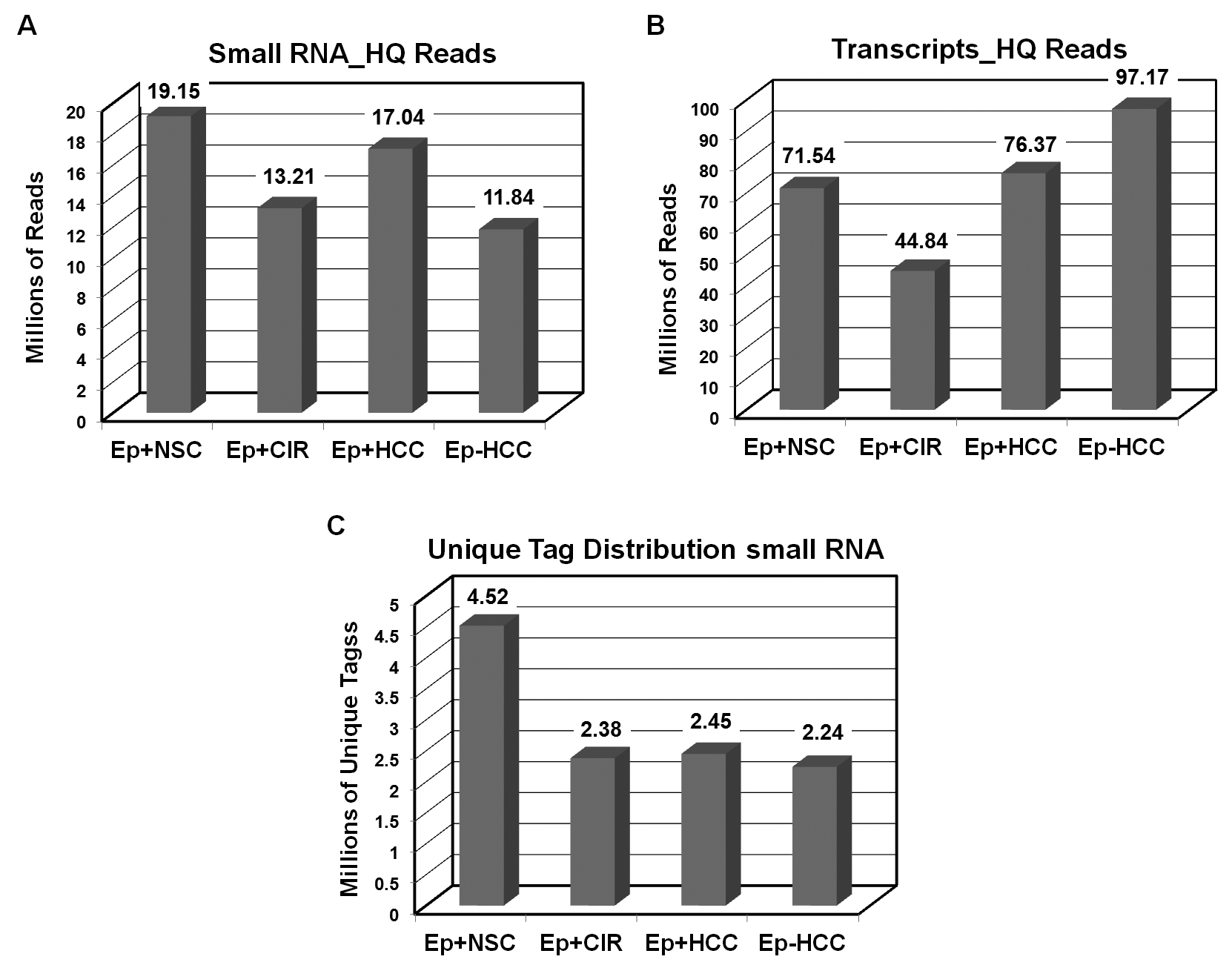
**

**Supporting Information Fig. 4.** (A and B) Number of reads of smallRNA and mRNA obtained in each sample. (C) Unique tag distribution of smallRNA observed in each sample.
