# Supplementary material for: EpCAM+ Liver Cancer Stem‐Like Cells Exhibiting Autocrine Wnt Signaling Potentially Originate in Cirrhotic Patients
Source: Stem Cells Transl Med. 2017 Jan 18;6(3):807–18. doi: 10.1002/sctm.16-0248 (PMC5442787; doi:10.1002/sctm.16-0248)
Supplement: Supplementary file 2 — Supporting Information Tables. [file SCT3-6-0807-s002.docx]

**Supporting Information Table 1. List of primer sequences used in qRT-PCR**

| **S. No** | **Gene** | **Primer sequence** |
| --- | --- | --- |
| 1 | *Nanog* | 5’-ACCAGACCTGGAACAATTCA-3’  5’-ATGCAGGACTGCAGAGATTC-3’ |
| 2 | *Oct4* | 5’-CTGTACTCCTCGGTCCCTTT-3’  5’-CCAGGTTTTCTTTCCCTAGC-3’ |
| 3 | *Sox2* | 5’-CGGTACGCTCAAAAAGAAAA-3’  5’-CCCAAAAAGAAGTCCAGGAT-3’ |
| 4 | *Wnt 3* | 5'-ACAGCCGGGGCAAGTTGGTAC-3'  5'-CCGGTGCTCTCATTGCGCAC-3' |
| 5 | *Cyclin D1* | 5'-CTGGAATCAGGGAACCCTACTC-3'  5'-GGACAGCAGAGCAGCCGTGAA-3' |
| 6 | *β-catenin* | 5'-GACAGCAATCAGCTGGCCTGGT-3'  5'-ACCACTCCCACCCTACCAACCA-3' |
| 7 | *Evi/Wls* | 5’-TCATGGTATTTCAGGTGTTTCG-3’  5’-GCATGAGGAACTTGAACCTAAA-3’ |
| 8 | *Notch 1* | 5’-CGGGTCCACCAGTTTGAATG-3’  5’-GTTGTATTGGTTCGGCACCAT-3’ |
| 9 | *Notch 2* | 5'-GTGCAGGAATTGGAAAGTTGG-3'  5'-GGCCGCTTCAGAGGAAAAG-3' |
| 10 | *Notch 3* | 5’-GCCATCTCCCTTTGGGAACT-3’  5’-CCACATTTACAGGGACATAAA-3’ |
| 11 | *Notch 4* | 5’-CCAAGAAATGCCCATAAACCAA-3’  5’-GCCTTTTAATGGGTAATCATTT-3’ |
| 12 | *Jag1* | 5’-CCAGGTCTTACTACGGAGCACA-3’  5’-CGCAAGCGATGTAGATTGAATA-3’ |
| 13 | *Hey2* | 5’-CTGTGCTCAGCGCAGCCGTC-3’  5’-TGAGCACAGAAAGTCATCAA-3’ |
| 14 | *18S* | 5’-AAGTACGCACGGCCGGTACA-3’  5’-AGCGCCCGTCGGCATGTATT-3’ |

**Supporting Information** **Table 2.** **Relative expression of genes determined by qRT-PCR**

| **Gene** | **Relative gene expression in** | | | | **p^#^** | **p^$^** | **p*** | **p^†^** |
| --- | --- | --- | --- | --- | --- | --- | --- | --- |
|  | **Ep+NSC (A)** | **Ep+CIR (B)** | **Ep+HCC (C)** | **Ep-HCC (D)** |  |  |  |  |
| *Nanog* | 0.9 | 3.4 | 3.5 | 2.3 | 0.000 | 0.006 | 0.930 | 0.068 |
| *Oct 4* | 0.5 | 2.4 | 2.4 | 1.0 | 0.005 | 0.014 | 0.862 | 0.030 |
| *Sox 2* | 0.4 | 3.0 | 2.7 | 0.9 | 0.006 | 0.000 | 0.672 | 0.023 |
| *Notch 1* | 0.3 | 1.4 | 1.6 | 0.9 | 0.025 | 0.028 | 0.745 | 0.047 |
| *Notch 2* | 0.5 | 2.0 | 2.2 | 2.1 | 0.016 | 0.016 | 0.868 | 0.589 |
| *Notch 3* | 0.4 | 2.0 | 2.6 | 1.2 | 0.015 | 0.008 | 0.410 | 0.041 |
| *Notch 4* | 1.0 | 1.4 | 2.1 | 1.1 | 0.257 | 0.649 | 0.284 | 0.11 |
| *Jag 1* | 0.7 | 2 | 2.6 | 1.9 | 0.015 | 0.042 | 0.275 | 0.407 |
| *Hey 2* | 0.6 | 2.6 | 2.8 | 2.0 | 0.062 | 0.124 | 0.633 | 0.116 |
| *Wnt 3* | 0.9 | 1.8 | 2.1 | - | 0.002 | 0.035 | 0.097 | - |
| *Evi/Wls* | 0.2 | 0.9 | 1.7 | - | 0.001 | 0.012 | 0.036 | - |
| *β-catenin* | 0.4 | 1.3 | 1.9 | - | 0.006 | 0.016 | 0.205 | - |
| *Cyclin D1* | 0.3 | 1.5 | 1.8 | - | 0.010 | 0.041 | 0.521 | - |
| *c-MYC* | 0.4 | 2.3 | 2.9 | - | 0.002 | 0.008 | 0.346 | - |

*p < 0.05 = significant*

*^#^ = between A & C; ^$^ = between A & B; ^*^ = between B & C; ^†^ = between C & D*

**Supporting Information Table 3. List of miRNAs specifically expressed in both Ep+CIR and Ep+HCC**

| **miRNA ID** | **Ep+NSC (FPKM)** | **Ep+CIR (FPKM)** | **Ep+HCC (FPKM)** | **Ep-HCC (FPKM)** |
| --- | --- | --- | --- | --- |
| hsa-miR-141-5p | 1.20 | 533.74 | 4.08 | 1.41 |
| hsa-miR-18a-3p | 1.20 | 2.28 | 5.17 | 1.78 |
| hsa-miR-200a-5p | 1.31 | 2511294.86 | 6.54 | 1.59 |
| hsa-miR-200b-5p | 1.57 | 1668360.93 | 9.32 | 1.41 |
| hsa-miR-210-5p | 1.20 | 8.88 | 18.77 | 1.59 |
| hsa-miR-224-3p | 1.43 | 5.17 | 2.89 | 1.78 |
| hsa-miR-31-5p | 1.31 | 16158.44 | 21.11 | 1.78 |
| hsa-miR-511-5p | 1.09 | 78.79 | 5.82 | 1.13 |
| hsa-miR-548o-3p | 1.43 | 2.60 | 2.03 | 1.41 |
| hsa-miR-561-5p | 1.20 | 10.20 | 26.72 | 1.13 |
| hsa-miR-7-1-3p | 1.20 | 3.92 | 2.57 | 1.41 |

*FPKM = Fragments Per Kilobase per Million mapped reads*

*FPKM >2 is considered to be expressed*
