# Supplementary material for: EpCAM+ Liver Cancer Stem‐Like Cells Exhibiting Autocrine Wnt Signaling Potentially Originate in Cirrhotic Patients
Source: Stem Cells Transl Med. 2017 Jan 18;6(3):807–18. doi: 10.1002/sctm.16-0248 (PMC5442787; doi:10.1002/sctm.16-0248)
Supplement: Supplementary file 4 — Supporting Information. [file SCT3-6-0807-s004.doc]

**EpCAM+ liver cancer stem-like cells exhibiting autocrine Wnt signalling potentially originate in cirrhotic patients**

Ritu Khosla, Archana Rastogi, Gayatri Ramakrishna, Vinayendra Pamecha, Ashok Mukhopadhyay, Madavan Vasudevan, Shiv Kumar Sarin, Nirupma Trehanpati

**SUPPORTING INFORMATION**

**PATIENTS AND METHODS**

**Small RNA Sequencing**

RNA isolated from one sample each of Ep+NSC, Ep+CIR, Ep+HCC and Ep-HCC was subjected to Illumina deep sequencing for small RNAs. Adapters were first ligated to the 5' and 3' ends of the total RNAs, and the ligated samples were used as the templates for cDNA synthesis. The cDNA was then amplified by polymerase chain reaction (PCR) to enrich the libraries. Following enrichment, the PCR products (~120–140 bp) were purified by polyacrylamide gel electrophoresis (PAGE) and their qualities and concentrations were confirmed with an Agilent 2100 Bioanalyzer (Agilent Technologies, CA, USA). The DNA fragments in the libraries were then used for cluster generation and sequencing on an Illumina HiSeq 2000 instrument (Illumia Inc., CA, USA).

**mRNA Sequencing**

Total RNA was purified using Sera-Mag Oligo (dT) beads (ThermoFisher Scientific, DE, USA) after DNase I treatment. The mRNA was fragmented by heating and then treating with sodium acetate. The cleaved RNA fragments were transcribed into first-strand cDNA using reverse transcriptase, followed by second-strand cDNA synthesis employing DNA polymerase and RNase H and cDNA purification using the QIAquick Gel Extraction kit (Qiagen, CA, USA). The double-stranded cDNA was further subjected to end repair employing T4 DNA polymerase, the Klenow fragment, and T4 polynucleotide kinase followed by a poly(A)-tailing procedure using Klenow exo-polymerase and ligation with an adapter using T4 DNA ligase. Adapter-ligated fragments were separated by agarose gel electrophoresis, and the desired range of cDNA fragments (200 ± 25 bp) was excised from the gel and enriched by PCR to construct the final cDNA library. After validation with Agilent 2200, the cDNA library was pair-end sequenced on a flow cell using Illumina HiSeq 2000.

**SmallRNA Seq data analysis**

Low quality reads were trimmed using NGSQC Tool kit [1]. After elimination of redundancy, sequences ≥ 18 nt were mapped to the human genome build HG19. Sequences that perfectly matched the genome along their entire length were considered for subsequent analyses. Genome sequences and annotations of the human genome (Hg19) were downloaded from NCBI. After alignment to the genome, reads were normalized calculating miRNAs per million miRNA alignments. A criterion that includes a read count >3, FPKM (fragments per kilobase of transcript per million mapped reads) >=1 and Log2ratio >=1 was considered for qualifying a miRNA as expressed in a sample. The list of significantly regulated miRNAs was created using a t-test analysis (p ≤ 0.05) and fold change (≥2.0) cut-off between two conditions.

**RNASeq data analysis**

Stringent quality control of Paired End sequence reads of all the samples was done using NGSQCTool kit [1]. Paired end sequence reads with Phredscore >Q20 was taken for further analysis. Human genome build Hg19 was used for SpliceVar read alignment and identification of transcripts that are expressed in both the libraries independently. TopHat pipeline [2] was used for alignment and Cufflink and Cuffdiff pipeline [3] was used for identification of transcript coding regions followed by quantitation and annotation using default parameters. Transcripts with an average read count of >=10, FPKM of >=1 and a log2ratio >=1 was considered as expressed. Sample specific transcript expression, baseline transcript expression and differential expression analysis (Fold change above 2 and p value <=0.05) was done based on the above criteria. Unsupervised hierarchical clustering of differentially expressed genes was done using Cluster 3.0 [4] and visualized using Java Tree View [5]. Statistically significantly enriched gene ontologies and pathways that harbour differentially expressed transcripts were identified using GOElite tool [6].

**Statistical Analysis and Differentially expressed transcripts**

Differentially expressed transcripts and miRNAs between patient groups were identified by DESeq data analysis pipeline using a fold-change threshold of absolute fold-change >=2 and a statistically significant Student’s t-test P value threshold adjusted for false discovery rate of less than 0.001. Statistically significantly enriched functional classes with a P value adjusted for false discovery rate of less than 0.05 derived using the hypergeometric distribution test corresponding to differentially expressed genes were determined using Student’s t-test with Benjamini Hocheberg FDR test. Unsupervised hierarchical clustering of differentially expressed genes between patient groups was done using Euclidian algorithm with Centroid linkage rule to identify gene clusters.

**miRNA Target Identification, Biological Pathways and Gene Ontology Enrichement Analysis**

Experimentally validated miRNA target genes information was obtained from miRTarbase (http://mirtarbase.mbc.nctu.edu.tw/). Differentially expressed transcripts and targets of differentially expressed miRNA were subjected for biological significance analysis by GOElite tool [6]. Complete human proteome was used as the background and gene sets were used as query. Database of GeneOntology categories, Wikipathways, KEGG Pathways, Pathway Commons, was configured for significance analysis. Each query list was subjected to over representation analysis against each of the above databases. A Z score and permutation or Fisher’s exact test p-value were calculated to assess over-representation of enriched biological categories.

**Integrome Analysis of differentially expressed genes and miRNA**

List of over represented biological categories identified in differentially expressed transcripts and differentially expressed miRNA targets were used for Integrome analysis. IntegroMatrix software (Bionivid Technology Pvt Ltd, Bangalore, India) was used to identify commonly over represented biological categories between patient groups at both gene level and miRNA target level. Commonly enriched biological categories were taken along with their statistical significance and differentially expressed genes and miRNA involved for downstream Integrome network analysis.

**SUPPORTING INFORMATION REFERENCES**

1. Patel RK, Jain M. NGS QC Toolkit: a toolkit for quality control of next generation sequencing data. **PLoS One***.* 2012;7:e30619.

2. Trapnell C, Pachter L, Salzberg SL. TopHat: discovering splice junctions with RNA-Seq. **Bioinformatics***.* 2009;25:1105-1111.

3. Trapnell C, Roberts A, Goff L et al. Differential gene and transcript expression analysis of RNA-seq experiments with TopHat and Cufflinks. **Nature protocols***.* 2012;7:562-578.

4. de Hoon MJ, Imoto S, Nolan J et al. Open source clustering software. **Bioinformatics***.* 2004;20:1453-1454.

5. Saldanha AJ. Java Treeview--extensible visualization of microarray data. **Bioinformatics***.* 2004;20:3246-3248.

6. Zambon AC, Gaj S, Ho I et al. GO-Elite: a flexible solution for pathway and ontology over-representation. **Bioinformatics***.* 2012;28:2209-2210.
